# A systematic review of questionnaires measuring asthma control in children in a primary care population

**DOI:** 10.1038/s41533-023-00344-9

**Published:** 2023-07-11

**Authors:** Sara Bousema, Arthur M. Bohnen, Patrick J. E. Bindels, Gijs Elshout

**Affiliations:** Department of General Practice, Erasmus MC, University Medical Centre Rotterdam, P.O. Box 2040, 3000 CA Rotterdam, The Netherlands

**Keywords:** Outcomes research, Asthma, Paediatric research

## Abstract

Several questionnaires are used to measure asthma control in children. The most appropriate tool for use in primary care is not defined. In this systematic review, we evaluated questionnaires used to measure asthma control in children in primary care and determined their usefulness in asthma management. Searches were performed in the MEDLINE, Embase, Web of Science, Google Scholar and Cochrane databases with end date 24 June 2022. The study population comprised children aged 5–18 years with asthma. Three reviewers independently screened studies and extracted data. The methodological quality of the studies was assessed, using the COSMIN criteria for the measurement properties of health status questionnaires. Studies conducted in primary care were included if a minimum of two questionnaires were compared. Studies in secondary or tertiary care and studies of quality-of-life questionnaires were excluded. Heterogeneity precluded meta-analysis. Five publications were included: four observational studies and one sub-study of a randomized controlled trial. A total of 806 children were included (aged 5–18 years). We evaluated the Asthma Control Test (ACT), childhood Asthma Control Test (c-ACT), Asthma APGAR system, NAEPP criteria and Royal College of Physicians’ ‘3 questions’ (RCP3Q). These questionnaires assess different symptoms and domains. The quality of most of the studies was rated ‘intermediate’ or ‘poor’. The majority of the evaluated questionnaires do not show substantial agreement with one another, which makes a comparison challenging. Based on the current review, we suggest that the Asthma APGAR system seems promising as a questionnaire for determining asthma control in children in primary care.

## Introduction

Asthma is a chronic pulmonary disease. It is characterized by wheezing, coughing, dyspnea and airway inflammation^1^. In children, asthma is the most prevalent chronic disease in primary care. The estimated prevalence of childhood asthma in Dutch primary care is 6.1%^2^ and prevalence in children is increasing^3^.

Asthma control is defined as the extent to which the effects of the disease can be seen in the patient, or have been reduced or removed by treatment^4,5^. It comprises two domains: symptom control and the future risk of adverse outcomes^6^. The assessment of asthma control is based on the presence of symptoms, limitations on activities and the use of rescue medication^6^. A significant proportion of pediatric asthma patients in primary care have suboptimal or uncontrolled asthma, which is associated with a decreased health-related quality of life (HRQL)^7,8^. It is important to determine asthma control because this provides insight into the burden of the disease and helps clinicians to decide on the best treatment strategy.

In addition to clinical tests such as spirometry, several questionnaires have been developed to measure asthma control. Frequently used instruments to measure asthma control in children are the Asthma Control Questionnaire (ACQ), the Asthma Control Test (ACT) and the Childhood Asthma Control Test (C-ACT). These questionnaires have been extensively evaluated, validated and compared in secondary and tertiary care settings^9–15^. A limited number of studies have been conducted in primary care, and these studies mainly focused on adults^15–18^.

In the primary care guidelines on pediatric asthma, asthma control as determined by the Global Strategy for Asthma Management and Prevention (GINA) guidelines^6^ is an important determinant of treatment^19^. It is important to use a tool that is reliable and easily identifies children with adverse outcomes who may benefit from a change in medication. Because General Practitioners (GPs) only have limited time available for each consultation, questionnaires should not take too much time to administer. Despite the numerous studies of asthma control tests, the most appropriate tool for use in primary care has not yet been identified.

In this review, we aim to compare the psychometric properties of asthma control questionnaires used in primary care. We compared these questionnaires regarding: 1) the symptoms and domains evaluated; 2) the characteristics of the questionnaires; 3) an assessment of their quality; 4) the agreement or correlation in their determination of asthma control; 5) the ability to detect uncontrolled asthma; and 6) the ability to predict future events. By evaluating these characteristics, we aim to determine the usefulness of these questionnaires in asthma management in children in primary care.

## Methods

### Identification and selection of the literature

A systematic literature search (without start date limitation and with end date 24 June 2022) was conducted in the MEDLINE, Embase, Web of Science, Google Scholar and Cochrane databases. In collaboration with a medical librarian specialized in literature searches, we searched for the elements ‘Asthma’, ‘Child’, ‘Questionnaires’ and ‘Comparison’ OR ‘Primary care’. These elements were converted into keywords (MeSH terms and Emtree terms) and words in the title and abstract. Case reports, conference abstracts, letters and editorials were excluded. No filter was used by language or date (see Supplementary Information file for the search documents). This trial was prospectively registered in the PROSPERO register under registration number CRD42019122793.

Titles and abstracts found using the search strategy were screened independently by three reviewers (SB, MR and AB). In the initial protocol we stated that we would include studies on children aged 6 years and older. However, because many studies use the age of 5 years as the lower limit of the age categories, we changed our lower age limit to 5 years. Papers were included for full-text analysis if they described a study in children with asthma (as defined in the criteria below), aged 5–18 years, in primary care. A minimum of two tools or questionnaires to determine asthma control had to be compared. Full papers were retrieved if the abstract provided insufficient information or if the paper met the criteria of the first screening. The reference lists of all the selected publications were checked for additional relevant publications. Disagreements were resolved by consensus or by consulting a fourth reviewer (GE). The extracted data included the setting, design, study population and outcome measures. We extracted information from the included studies on the development and the purpose of the questionnaires. We derived information on the ability to detect uncontrolled asthma from the included studies. If no information was available, we searched for the information in the initial validation study of the questionnaire. These studies could also be conducted in secondary or tertiary care.

The methodological quality was assessed by two independent reviewers using the COSMIN quality criteria for the measurement properties of health status questionnaires, which are based on international consensus^20^. Questionnaires were scored for the following domains (when applicable): content validity, internal consistency, criterion validity, construct validity, reproducibility (agreement and reliability), responsiveness, floor and ceiling effects and interpretability.

Studies had to meet the following criteria:

- The study design was a randomized controlled trial, cross-sectional study, prospective cohort or study with case-control questionnaires (self-administered, parent-administered and interviewer-administered questionnaires were all included).
- The participants were children aged 5–18 years with a confirmed diagnosis of asthma. Asthma was defined as satisfaction of one or more of the following criteria:
  - Doctor’s diagnosis of asthma (‘clinician-diagnosed asthma’).
  - Coded as having a diagnosis of asthma (e.g. International Classification of Primary Care (ICPC) read codes).
  - More than one of the following symptoms: wheezing, breathlessness, chest tightness, cough, reversibility of FEV1 > 12% in spirometry.
  - The use of inhaled corticosteroids (ICS) because of asthma symptoms.

- The study was conducted in primary care or in a primary care population.
- The article was in English, Dutch or Spanish.
- The outcome measures were of the following types: results of validated questionnaires in children describing asthma control, or asthma control measured by a tool developed by a national guideline organization. Both structured tools (e.g. C-ACT) and unstructured tools (e.g. VAS) were included.
- Studies with children and adults were included if a subgroup analysis was conducted for children below 18 years.

Studies were excluded if they:

- Described questionnaires to measure asthma control in terms of quality of life.
- Compared different ways to administer a questionnaire to measure asthma control (for example: interviewer version vs. written questionnaire vs. electronic questionnaire, or parent vs. child).
- Compared one single questionnaire with a clinical test for measuring asthma control (such as spirometry or fractional nitric oxide concentration measurements in exhaled breath).

The extracted information included: 1) the symptoms and domains evaluated; 2) the characteristics of the questionnaires; 3) a quality assessment; 4) the agreement or correlation in determination of asthma control; 5) the ability to detect uncontrolled asthma and thereby identify the children who would benefit from a change in therapy; 6) the ability to predict future events.

### Reporting summary

Further information on research design is available in the Nature Research Reporting Summary linked to this article.

## Results

**Fig. 1:**
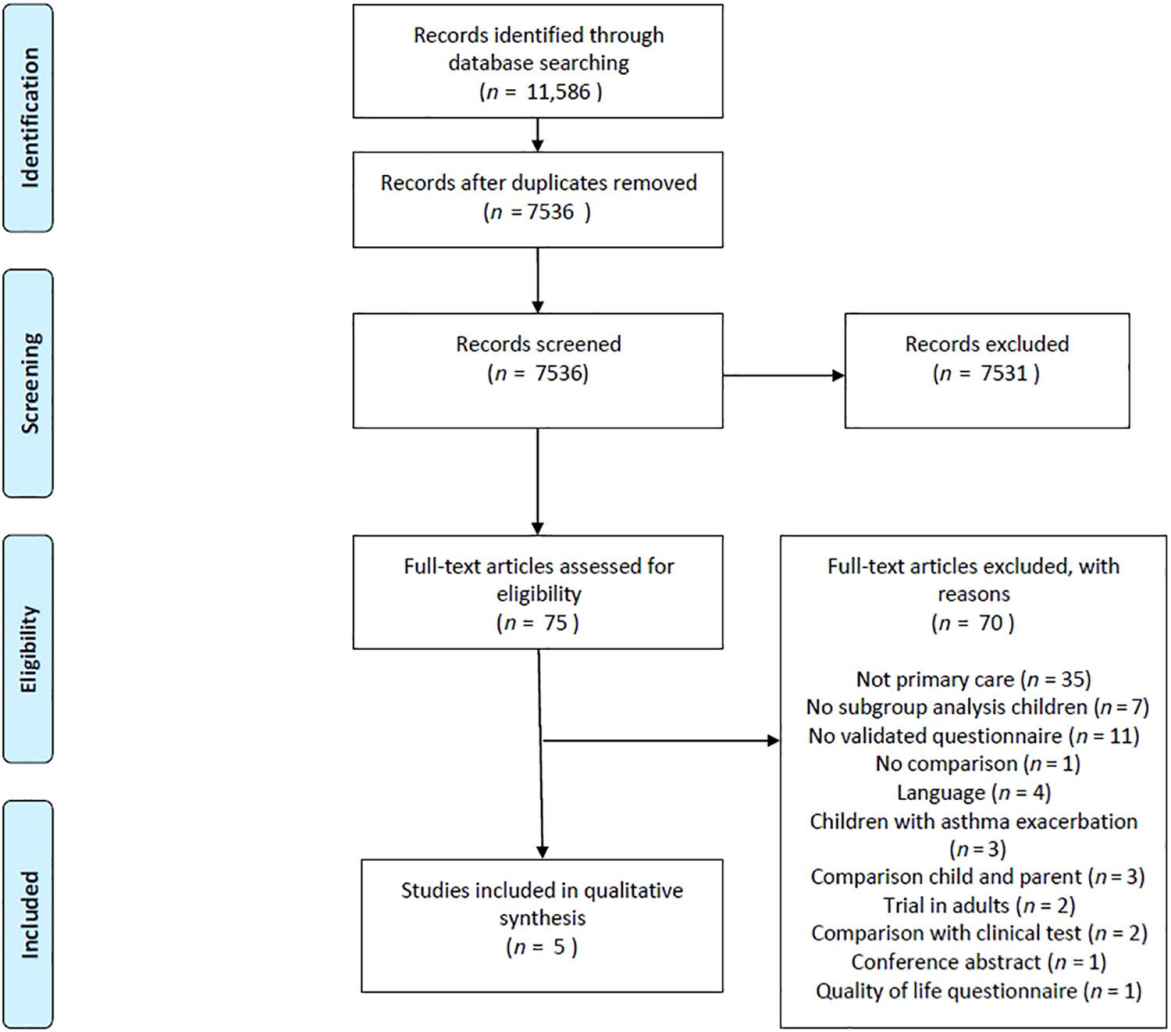
Flowchart of the process of identification and inclusion of studies.

The search strategy yielded 7536 records, of which 75 were eligible for inclusion based on the title and abstract. The remaining 7461 records were excluded for various reasons, e.g. the study did not concern asthma, the study concerned the treatment of adult asthma patients or the study evaluated quality-of-life questionnaires. We screened full-text versions of the 75 eligible articles. Five publications were ultimately included. Figure 1 shows a PRISMA flowchart of the process of identification and inclusion of studies for the current review.

The five studies selected for this review had a total of 1085 participants (range 35–468). The age of the participants varied from 5 to 71 years. A total of 806 children were included in the studies. Four studies had an observational design. The fifth study, by Rank et al., was a sub-study of a randomized controlled trial. Three studies were conducted in the United Kingdom (UK)^21–23^ and two in the United States of America (USA)^24,25^. Two studies also included adult patients (55.3%^24^ and 57.1% respectively)^21^; however, subgroup analyses were conducted. Rank et al. conducted their study in twenty^24^ primary care practices and Andrews et al. in eight^23^ primary care practices. Halterman et al. recruited patients from three urban clinics and three suburban practices (in the discussion referred to as ‘primary care offices’)^25^. The participants in the study by Thomas et al. were recruited from nurse-led asthma clinics at two general practices^21^. Juniper et al. included children from five primary care sites and one hospital clinic^22^. Asthma was defined as ‘clinical diagnoses of asthma’^23^, ‘documented evidence of asthma’^21^ or ‘physician-diagnosed’ asthma^24^. Halterman et al. included all children who had a diagnosis of asthma and had >2 asthma-related visits in the prior 12 months^25^. In the study of Juniper et al., children were eligible if they had well-established and physician-diagnosed asthma, with current symptoms of asthma (ACQ score > 0.5)^22^. The questionnaires (VAS and NEAPP) were filled in by the parents in the study by Halterman et al.^25^. In the study by Thomas et al.^21^, the questionnaires were administered by a clinician. In the other three studies the questionnaires were filled in by the children (sometimes together with their parents)^22–24^. Tables 3 and 5 show this information and the main results of the included studies. The studies included in this review were considered to be too heterogeneous (in terms of the questionnaires used, setting and patient categories) to pool the data. Table 1 shows the characteristics of the included studies.

**Table 1:** Characteristics of the included studies.

Reference	Year	Country	Design	Questionnaires	*N*	Age
Andrews	2018	UK	Observational study	**RCP3Q**, ACT, C-ACT	319	5–16
Rank	2014	USA	Sub-study of randomized controlled study	**APGAR**, ACT, C-ACT	468^a^	5–45
Juniper	2010	UK	Observational study	**ACQ**, RCP3Q, *ACD*	35	6–16
Thomas	2009	UK	Observational study	**RCP3Q**, ACQ	35^b^	6–71
Halterman	2006	USA	Observational study	NAEPP, VAS	228	5–12

The questionnaire of primary interest is marked in bold.

*RCP3Q* Royal College of Physicians’ ‘three questions’, *ACT* Asthma Control Test, C-ACT Childhood Asthma Control Test, *APGAR* Activities Persistent triGGers Asthma medications Response to therapy, *ACQ* Asthma Control Questionnaire, ACD: Asthma Control Diary (not validated in children), *NAEPP* National Asthma Education and Prevention Program, *VAS* Visual Analog Scale.

^a^209 children in the total sample of 468 (=44.7% were aged ≤18 years).

^b^15 children in the total sample of 35 (=42.9% were aged ≤18 years).

### Measurement characteristics of the questionnaires

The five studies presented results of the comparison of two or more questionnaires for measuring asthma control^21–25^. These studies gave comparisons of the following structured and unstructured questionnaires: Asthma Control Diary (ACD), ACT, ACQ, C-ACT, National Asthma Education and Prevention Program (NAEPP) criteria, Royal College of Physicians’ ‘three questions’ (RCP3Q), Visual Analog Scale (VAS) and the Asthma APGAR system (APGAR is an acronym for Activities, Persistent, triGGers, Asthma medications and Response to therapy). The ACD is evaluated in the study by Juniper et al. ^22^. Since the ACD has not been validated in children, we do not describe this tool.

### Comparison of questionnaires

#### Symptoms and domains evaluated

Each questionnaire deals with a different combination of symptoms and domains. Table 2 shows the domains covered.

**Table 2:** Symptoms and domains covered by questionnaires to assess asthma control.

	ACT	ACQ	APGAR	C-ACT	NAEPP	RCP3Q	VAS
Daytime symptoms		x	x	x	x	x	NA
Limitations on activities	x	x	x	x	x	x	NA
Lung function		x					NA
Shortness of breath/chest tightness	x	x	x				NA
Nocturnal symptoms	x	x	x	x	x	x	NA
Patient’s perception of control	x						NA
Overall symptoms			x				NA
Wheezing		x		x			NA
Coughing			x	x			NA
Triggers			x				NA
(Rescue) β-agonist use	x	x	x		x		NA
Effectiveness of reliever medication			x				NA
Exacerbations^a^					x		NA

*ACT* Asthma Control Test, *ACQ* Asthma Control Questionnaire, *APGAR* Activities Persistent triGGers Asthma medications Response to therapy, *C-ACT* Childhood Asthma Control Test, *NAEPP* National Asthma Education and Prevention Program, *RCP3Q* Royal College of Physicians’ ‘three questions’, *VAS* Visual Analog Scale

^a^Requiring oral systemic corticosteroids.

### Characteristics of the questionnaires

#### Asthma Control Questionnaire

The ACQ score is the mean of seven questions and ranges between 0 (totally controlled) and 6 (severely uncontrolled). The last question of the ACQ concerns the value of FEV1 and is filled in by a clinician. The ACQ has been validated for children aged 11 years and older^26–28^. For children aged 6–10 it must be administered by a trained interviewer^10^. Three shortened versions of the ACQ have been validated as well, but the complete ACQ has the strongest measurement properties^28^.

#### Asthma Control Test

The ACT is a self-administered questionnaire for children aged 12 years and up. It contains five items^29^.

#### Childhood-Asthma Control Test

The C-ACT is a seven-item questionnaire that has three questions for parents and four questions for children. It has been validated in children aged 4–11 years^13^.

#### Asthma APGAR system

The Asthma APGAR system has recently been developed for use in a primary care population^30^. It was developed to be answered by both parents and children together. After they have completed the assessment, an algorithm based on that data guides the clinicians in their treatment strategy for the patient. The score that corresponds to inadequate asthma control is derived from the National Asthma Education and Prevention Program (NAEPP) guidelines^31^.

#### NAEPP criteria

The NAEPP guideline-based criteria to assess asthma control are part of the National Asthma Education and Prevention Program in the USA^32^. This expert panel organization emphasizes the importance of monitoring asthma control. The level of severity is determined by assessing both impairment and risk. Asthma control is determined per age category (0–4 years, 5–11 years and ≥12 years).

#### Royal College of Physicians’ ‘three questions’

The Royal College of Physicians in the UK has developed a practical clinical tool containing three questions (RCP3Q) to assess asthma control in primary care^33^. It is the most commonly used tool in the UK. The questionnaire was designed by primary and secondary care physicians and patient organizations. It was designed to be completed by a health-care professional and contains three questions with the answer options ‘Yes’ or ‘No’, with a score of 1 for ‘yes’ and 0 for ‘no’. The total score ranges between 0 and 3. An RCP3Q score of 0 indicates good asthma control and a score of 2 or 3 indicates poor control^34^. The UK Quality Outcomes Framework (QOF)^35^ encourages the use of the RCP3Q in patients aged 8 and older. The performance of this questionnaire has been evaluated in adults; however, there is limited evidence for the use in children.

#### Visual Analog Scale

The VAS is an unstructured method for assessing asthma control in patients. To determine the VAS score, patients (or parents) have to indicate the severity of symptoms by placing an ‘X’ along a 100 mm line. A score of 0 (X on the left) indicates ‘no symptoms’ and a score of 100 (X on the right) indicates ‘very bad symptoms’. The VAS score is collapsed into quartiles (0–25, 26–50, 51–75, 76–100) corresponding to an ascending level of asthma severity. No cut-off value has been described.

Table 3 shows the characteristics of the questionnaires for assessing asthma control that are included in the current review. Table 4 gives information on the development and the purpose of the questionnaires.

**Table 3:** Characteristics of questionnaires for assessing asthma control.

	Age (years)	Recall period	Number of items	Range	Cut-off point for uncontrolled asthma
ACT	≥12	4 weeks	5	5-25	≤19
C-ACT	4–11	4 weeks	7	0-27	≤19
ACQ	>6^a^	1 week	7^b^	0 = well controlled 6 = extremely poorly controlled	1.5
APGAR	5–18	2 weeks	6		A + P ≥ 2
NAEPP	0–18	2–4 weeks	4	well controlled not well controlled very poorly controlled	NA
RCP3Q	8	1 month	3	0–3	≥1
VAS		3 months^c^	1	0–100 0 = no symptoms 100 = very bad symptoms	

*ACT* Asthma Control Test, *C-ACT* Childhood Asthma Control Test, *ACQ* Asthma Control Questionnaire, *APGAR* Activities Persistent triGGers Asthma medications Response to therapy, *NAEPP* The National Asthma, Education and Prevention Program, *RCP3Q* Royal College of Physicians’ ‘three questions’, *VAS* Visual Analog Scale.

^a^ACQ: in children aged 6–10 years, it must be administered by a trained interviewer.

^b^Shortened versions of the ACQ exist.

^c^In the trial of Halterman et al.

**Table 4:** Development and purpose of the questionnaires.

	Developed by	Purpose
ACT	Primary care clinicians/ leading asthma specialists	Brief patient-based assessment of asthma control
C-ACT	Asthma specialists	To assess asthma control in children aged 4–11 years with asthma, for use in the clinic and at home (self-administered)
ACQ	Clinicians (members of international asthma guideline committees)	To measure asthma control as defined by international guidelines, minimize symptoms and reduce the risk of exacerbations
APGAR	Primary care clinicians	To address the gap in the primary care management of asthma
NAEPP	NAEPP expert panel developed clinical guidelines for the diagnosis and management of asthma	No information
RCP3Q	Consensus from a multidisciplinary seminar	Practical tool to assess asthma control in primary care
VAS	Designed to document the characteristics of disease-related symptom severity in individual patients.	Unstructured method of estimating disease severity; rapid, statistically measurable and reproducible classification of symptom severity and disease control

### Quality assessment

Information on content validity could be derived from two studies^22,24^. No information was found on internal consistency. Criterion validity was evaluated in three studies^23–25^. Two studies evaluated construct validity^21,22^. The aspects of agreement, reliability and responsiveness were evaluated in one study^22^. Floor and ceiling effects were rated as poor in three studies^21,23,25^. All studies scored ‘intermediate’ on interpretability^21–25^. Table 6 in the supplementary information file gives a summary of the assessment of the measurement properties of all the questionnaires included in this review.

### Agreement in the determination of asthma control

The study by Andrews et al. determined the accuracy of the RCP3Q score in predicting asthma control as defined by the ACT or C-ACT threshold score of 19^23^. For children aged 5–11, a kappa value of 0.43 for poorly controlled asthma was found, indicating moderate agreement. For children aged 12–16, the kappa value was 0.33, demonstrating fair agreement. Overall, RCP3Q scores correlated moderately with C-ACT and ACT data (Spearman’s rho correlation coefficient was −0.52 and −0.49 respectively). Table 5 shows the agreement between the questionnaires included in the current review. The legend in Table 5 gives the interpretation of the kappa values and correlation values. The study showed that the RCP3Q’s sensitivity for detecting uncontrolled asthma as defined by ACT of C-ACT ranged from 43% to 60% and the specificity from 80% to 82%.

**Table 5:** Agreement of questionnaires in the included studies.

Reference	Questionnaires	Administered by	Main results
Andrews	RCP3Q versus ACT/C-ACT	ACT and RCP3Q: children C-ACT: children and parents	5–11 years RCP3Q threshold score 0 (well controlled asthma); kappa = 0.39 RCP3Q threshold score ≥2 (poorly controlled asthma); kappa = 0.43 12–16 years RCP3Q threshold score 0 (well controlled asthma); kappa = 0.26 RCP3Q threshold score ≥2 (poorly controlled asthma); kappa = 0.33
Rank	APGAR versus ACT/C-ACT	ACT: children APGAR: children and parents	5–11 years kappa = 0.716 12–18 years kappa = 0.625
Juniper	ACQ versus RCP3Q	ACQ, children and parents RCP3Q: clinician	Cross-sectional PCC^b^ = 0.52 Longitudinal PCC = 0.81
Thomas	RCP3Q versus ACQ	Clinician	Cross-sectional correlation coefficient; 0.41 (*p* = 0.134) Longitudinal correlation coefficient; 0.61 (*p*-value < 0.001)
Halterman	NAEPP^a^ versus VAS	VAS: parents NEAPP: parents	VAS M-I (%) M-P M-S-P (%) 0–25 76.4 39.5 8.0 26–50 23.6 44.2 33.3 51–75 0 14.0 34.5 76–100 0 2.3 24.1

Interpretation of kappa values: <0: less than chance agreement; 0.01–0.20: slight agreement; 0.21–0.40: fair agreement; 0.41–0.60: moderate agreement; 0.61–0.80: substantial agreement; 0.81–0.99: almost perfect agreement^50^.

Interpretation of correlation coefficients: 0.00–0.30: negligible correlation; 0.30–0.50: low positive correlation; 0.50–0.70: moderate positive correlation; 0.70–0.90: high positive correlation; 0.90–1.00: very high positive correlation^51^.

*M-I* Mild, intermittent, *M-P* Mild, persistent, *M-S-P* Moderate-severe, persistent, *ACD* Asthma Control Diary (not validated in children), *ACT* Asthma Control Test, *C-ACT* Childhood Asthma Control Test, *ACQ* Asthma Control Questionnaire, *APGAR* Activities Persistent triGGers Asthma medications Response to therapy, *NAEPP* The National Asthma Education and Prevention Program, *RCP3Q* Royal College of Physicians three questions, *VAS* Visual Analog Scale.

^a^This definition was used as the gold-standard assessment of severity.

^b^Pearson’s correlation coefficient.

Juniper et al. evaluated the measurement properties of the ACQ by comparing the results with the RCP3Q in 35 children^22^. Pearson correlation coefficients between the ACQ and the RCP3Q were determined. The value for cross-sectional construct validity was 0.52 and the value for longitudinal construct validity was 0.81.

Thomas et al. determined the correlation between the RCP3Q and the ACQ in adults and children. Fifteen children completed seven follow-up visits (over 12 weeks). The cross-sectional correlation coefficient in children was 0.41, however this moderate correlation was not statistically significant. The longitudinal correlation for children was 0.61 (*p* < 0.001). This study was an exploratory analysis.

Rank et al. tested the effectiveness of the Asthma APGAR system by comparing this questionnaire with the ACT and C-ACT^24^. A total of 209 participants in the overall study population were aged under 18 years (=44.7%). For children aged 5–11 years, the C-ACT and Asthma APGAR instruments were in agreement in 85.8% of the cases (95% CI 78.5–91.4%). The kappa value of 0.716 (95% CI: 0.060–0.84) indicated substantial agreement. In the age group 12–18 years, the two questionnaires were in agreement 81.3% of the time (95% CI 71.0–89.1%). The kappa value of 0.625 (95% CI: 0.45–0.80) indicated substantial agreement as well.

Halterman et al. compared the assessment of asthma control using NAEPP criteria with a VAS. The NAEPP severity classification was used as a gold standard. Both questionnaires were filled in by the parents. A critical error was defined as ‘if parents reported the child’s symptoms in the lower 50^th^ percentile of severity for VAS, whereas the child had moderate or severe persistent symptoms according to the NAEPP criteria’. The results showed that 41% of the parents made this so-called ‘critical error’.

### Ability to detect uncontrolled asthma

#### ACT

The screening accuracy of the ACT was evaluated by Nathan et al. ^29^. The agreement between the ACT and a specialist’s rating of asthma control was determined. A cut-off point of ≤19 resulted in a sensitivity of 69.2% and specificity of 76.2%, with an area under Receiver operating characteristic (ROC) curve of 0.727.

#### C-ACT

The validation study of the C-ACT by Liu et al.^13^ compared the C-ACT scores with a specialist’s assessment. It found that a cut-off point of 19 results in a sensitivity of 68% and a specificity of 74% for the detection of uncontrolled asthma.

#### ACQ

The study by Juniper et al. showed that in children whose asthma control changes between clinic visits, the questionnaire was able to detect the change (*p* < 0.026)^22^. However, no specific information can be extracted on the ability of the ACQ to detect uncontrolled asthma. The previous validation study in adults did not provide this information either^26^.

#### APGAR

No detailed information about the ability to detect uncontrolled asthma of the Asthma APGAR system can be found in the study of Rank at al. ^24^. The authors did identify an ‘actionable item’ in more than 75% of the children with poor asthma control.

#### NAEPP

The NAEPP and ACQ criteria were compared in a study of 373 adolescents with asthma. The NAEPP identified 84.6% of the cases of uncontrolled asthma and the ACQ 64.6% of the cases^11^.

#### RCP3Q

To analyze the performance of the RCP3Q in detecting uncontrolled asthma, it was compared to C-ACT or ACT, whereby a score of 19 was defined as uncontrolled asthma^23^. Using a threshold RCP3Q score of ≥2 to predict uncontrolled asthma resulted in a sensitivity of 0.60 and a specificity of 0.82 for the age group 5–11 years, and a sensitivity of 0.51 and specificity of 0.81 for the age group 12–16 years.

#### VAS

Halterman compared unstructured assessments of asthma severity (VAS) with the NAEPP classification of severity. Of the children with moderate to severe symptoms according to the NAEPP classification, 41% of the parents rated their children in the lowest two quartiles of the VAS. The unstructured method seems to underestimate the severity level of asthma.

### Ability to predict future events

None of the questionnaires included in this review provides information on the risk of future events as an outcome. Previous studies have identified several risk factors for asthma attacks or poor asthma-related outcomes^36–38^. These risk factors include e.g. younger age, history of hospitalization or an emergency department (ED) visit in the previous year, three days’ use of oral corticosteroids in the previous three months, a lower FEV1/FVC ratio^37^, higher FeNO levels and a recent history of asthma attacks^38^. A recent systematic review concluded that a previous asthma attack was the most strongly predictive factor^36^.

## Discussion

This is the first systematic review that evaluated the usefulness of pediatric asthma control questionnaires in a primary care population. Five studies were included. A ‘gold standard’ or reference standard to determine asthma control in children is lacking. The majority of the evaluated questionnaires do not show substantial agreement with other questionnaires, which makes a comparison challenging. The studies varied in the asthma definition used, the administration of the questionnaires (by the parents and/or child and clinician), method of statistical analysis, age range, included domains of asthma control and sample size. Moreover, there were differences in the recall period.

Several characteristics make a questionnaire suitable for use in clinical practice. A convenient asthma control questionnaire for children needs to be relatively quick to complete, able to identify patients with uncontrolled asthma at risk of adverse outcomes and able to identify patients who would benefit from a different treatment strategy.

Based on our evaluation, we conclude that the asthma APGAR system is a promising tool to determine asthma control in children in primary care. It is specifically designed for use in primary care. It shows good agreement with the validated C-ACT, and no spirometry results are needed to complete the questionnaire. It may be more time consuming to fill in than the other questionnaires; however, it includes an algorithm that guides the physicians in their management strategy, which could be more efficient. There is evidence that the introduction of the Asthma APGAR system improves rates of asthma control and reduces asthma-related ED visits, urgent care and hospital visits^39^. Since the Asthma APGAR system was only developed recently, it needs further validation before it can be implemented in pediatric asthma management in primary care.

Worth et al. conducted a systematic literature review to identify Patient-Reported Outcome Measures (PROMs) for asthma in adults and children^40^. The aim of the study was to identify PROMs for use in research contexts and clinical settings. For children, the only tools included in this review to determine asthma control were the Childhood Asthma Questionnaire (CAQ) and the C-ACT. The reviewers only included ‘sufficiently well developed and validated questionnaires’. In addition, the results were not specifically for a primary care population. The authors conclude that the CAQ is poorly validated and the C-ACT requires further validation work as there are doubts as to whether it estimates poor control of asthma accurately. The evaluated questionnaires show little overlap with the instruments in our study. Another literature review by Voorend-van Bergen et al. explored the usefulness of questionnaires commonly used to determine asthma control in children^41^. It described the measurement characteristics of the ACT, C-ACT and ACQ as well as the Asthma Therapy Assessment Questionnaire (ATAQ) and the Test for Respiratory and Asthma Control in Kids (TRACK). The authors did not conduct a systematic literature search; they merely described commonly used questionnaires. Besides, the study population was not restricted to primary care. The authors conclude that these tools to determine asthma control may be useful in pediatric asthma management, but they emphasize the need for validation studies in a wider range of settings. No particular questionnaire is recommended.

Measurement characteristics of the RCP3Q questionnaire were described in three articles included in the current review^21–23^. The correlation with other questionnaires in these studies varied from fair to good. Hoskins et al. assessed the diagnostic performance of the RCP3Q in patients aged ≥13 in primary care using statistical modeling and found that the RCP3Q model provided the best fit. 11% of the subjects were aged 13–19 years. The study was not included in this review because data for children were not presented separately. The results indicate that the RCP3Q is an effective tool for assessing asthma control in routine review consultations^42^. However, since these results concern both adults and children and no subgroup analysis was conducted, it is not clear whether these results apply specifically for children aged 18 years and younger.

The Visual Analog Scale is not widely used in asthma care, but the authors of a previous trial concluded that it could be an effective additional tool in the diagnostic process in children with exercise-induced asthma (EIA)^43^. Moreover, a prospective study assessed the value of VAS as a daily monitoring tool in 42 adolescents with asthma^44^. Patients were recruited from the emergency department through clinical referrals and with flyers. The authors conclude that the VAS score significantly predicted the results of symptom diary data. These two findings are not in accordance with the results of Halterman et al.^25^. One reason for this discrepancy could be the fact that patients in these two studies were recruited from the pediatric emergency department^44^ and from an outpatient clinic of the pediatric department^43^. It seems reasonable to assume that asthma control in children in secondary care is not comparable to asthma control in a primary care population. Besides, the method for administering the VAS score was different. Lastly, the study of Lammers et al. only included a specific subgroup of children, namely children with EIA.

Although no gold (or reference) standard exists to measure asthma control in children, the GINA criteria are sometimes referred to as such. The performance of the ACT and ACQ has been compared with the GINA criteria in children in multiple studies^9,45,46^. These studies all concerned hospital patients with asthma. Koolen et al. concluded that both the ACT and C-ACT underestimated the proportion of children with uncontrolled asthma as defined by GINA^9^. The trial by Yu et al. suggests that C-ACT scores and GINA guideline−based asthma control measures were positively correlated, but that the C-ACT may overestimate asthma control^46^. O’Byrne et al. used the GINA criteria as a gold standard to determine the accuracy of the ACQ-5^45^. They found a moderate correlation (kappa value 0.59) for children aged <18 years. None of the studies included in the current review compared a questionnaire with the GINA guidelines. Since the outcome of the GINA instrument is partly based on spirometry results, this could have influenced the level of agreement between the questionnaires.

The different questionnaires were designed with different purposes and outcomes in mind. The choice of questionnaire to use depends on the intellectual level of the child and parents and the age of the child. In Dutch primary care, spirometry results are not always available for children, since spirometry in children is not routinely carried out by GPs. Consequently, questionnaires that take into account spirometry, e.g. the ACQ, GINA and NAEPP criteria, are less appropriate. Alternative tools are the ACT, C-ACT, Asthma APGAR system or shortened versions of the ACQ. The ACT and C-ACT have been extensively validated in secondary care and are easy to administer^13,14,47^ The C-ACT displays pictures with facial expressions to represent emotions in relation to the answers. This could make this questionnaire more appealing and easier to complete for children. The C-ACT contains questions for both the parents and the child. The result of this questionnaire gives an integral assessment of asthma severity. However, previous trials were only conducted in secondary and tertiary care and suggest that C-ACT may overestimate asthma control^9,46^. This could lead to under-treatment, which negatively influences the diagnostic and therapeutic process of pediatric asthma patients. The Asthma APGAR system was designed for use in primary care. It shows good agreement with the validated ACT and C-ACT and it includes an algorithm for treatment.

There are some limitations to this study. First, the quality of most of the studies included in this review was rated ‘intermediate’ or ‘poor’ (when information was available) rather than ‘positive’. Secondly, not all the questionnaires currently used to determine asthma control in children are represented in this review. Commonly used tools such as GINA^6^ and the asthma therapy assessment questionnaire (ATAQ)^48^ are not described in the current review since there were no comparative studies of these tools performed in children in primary care. We decided to only include studies with full text in English, Dutch or Spanish, which may have resulted in the exclusion of articles that fulfilled our other inclusion criteria. One of the strengths of this study is the sensitive search strategy. This resulted in a large number of references. However, only a small number of studies were included. This reflects the strict inclusion criteria we used to select studies. Studies were only included if it was explicitly stated that the study was conducted in primary care. Furthermore, studies conducted in children and adults were excluded if no subgroup analysis was performed in children aged under 18. Children younger than 5 years were not included because the diagnosis of asthma is difficult to confirm in pre-school children^49^.

The present systematic review evaluated five studies that compared questionnaires used to determine asthma control. Based on the available evidence, we suggest that the Asthma APGAR system could be a promising tool for use by GPs in clinical practice. However, it needs further validation. More studies are needed to develop a questionnaire that assesses the risk of future events.

## Supplementary information

Supplementary Information

Reporting Summary

## Data Availability

The authors declare that the data supporting the findings of this study are available within the paper and in the supplementary information file.
