# Supplementary material for: A systematic review of questionnaires measuring asthma control in children in a primary care population
Source: NPJ Prim Care Respir Med. 2023 Jul 11;33:25. doi: 10.1038/s41533-023-00344-9 (PMC10336001; doi:10.1038/s41533-023-00344-9)
Supplement: Supplementary file 1 — Supplementary Information [file 41533_2023_344_MOESM1_ESM.pdf]

Search documents

	references	References after deduplication
embase.com	2801	2767
Medline Ovid	3603	1886
Web of science	2817	1415
Cochrane CENTRAL	490	261
Google scholar (200 top ranked)	200	105
**Total**	**9911**	**6434**

embase.com

('asthma'/exp OR 'Asthma Control Questionnaire'/de OR 'Asthma Control Test'/de OR (asthma*):ab,ti) AND (child/de OR pediatrics/exp OR childhood/exp OR 'child health'/de OR 'child health care'/exp OR 'child care'/exp OR 'childhood disease'/exp OR 'pediatric ward'/de OR 'pediatric hospital'/de OR (child* OR kid OR kids OR schoolchild*):ab,ti) AND ('questionnaire'/exp OR 'assessment of humans'/exp OR 'clinical assessment tool'/de OR 'patient assessment'/de OR (questionnaire* OR Control-Test* OR gina OR global-initiative* OR checklist* OR check-list* OR screening-tool* OR act OR acq OR instrument* OR scoring OR scale OR ((measure* OR assess* OR determine* OR tool*) NEAR/3 control*)):ab,ti) AND (('comparative study'/de OR 'comparative effectiveness'/de OR 'device comparison'/de OR 'intermethod comparison'/de OR (((compar*) NEAR/10 (questionnaire* OR Test* OR gina OR checklist* OR check-list* OR tool* OR method* OR intermethod* OR stud* OR assess* OR act OR acq OR instrument* OR scoring OR scale OR global-initiative* OR effect*)) OR (clinical* NEAR/3 value*) OR (measurement* NEAR/3 (propert* OR characterist*))):ab,ti) OR ('primary health care'/exp OR 'general practitioner'/de OR 'general practice'/de OR 'family medicine'/de OR 'Family Medicine':ad OR (((primary OR family OR general) NEAR/3 (care OR pract* OR medicine* OR physician* OR doctor*)) OR gp OR gps):ab,ti)) NOT ([Conference Abstract]/lim OR [Letter]/lim OR [Note]/lim OR [Editorial]/lim) NOT ('case report'/de OR ('case report*'):ab,ti)

Medline Ovid

(exp asthma OR (asthma*).ab,ti.) AND (exp Child/ OR exp Infant/ OR exp Adolescent/ OR exp "Pediatrics"/ OR exp "Child Health Services"/ OR exp "Child Care"/ OR "Hospitals, Pediatric"/ OR (child* OR kid OR kids OR schoolchild*).ab,ti.) AND ("Surveys and Questionnaires"/ OR (questionnaire* OR Control-Test* OR gina OR global-initiative* OR checklist* OR check-list* OR screening-tool* OR act OR acq OR instrument* OR scoring OR scale OR ((measure* OR assess* OR determine* OR tool*) ADJ3 control*)).ab,ti.) AND ((Comparative Study/ OR Comparative Effectiveness Research/ OR (((compar*) ADJ10 (questionnaire* OR Test* OR gina OR checklist* OR check-list* OR tool* OR method* OR intermethod* OR stud* OR assess* OR act OR acq OR instrument* OR scoring OR scale OR global-initiative* OR effect*)) OR (clinical* ADJ3 value*) OR (measurement* ADJ3 (propert* OR characterist*))).ab,ti.) OR (exp Primary Health Care/ OR primary care.af. OR exp Physicians, Family/ OR general pract$.af. OR family.in. OR family pract$.af. OR family physician$.mp.)) NOT (letter* OR news OR comment* OR editorial* OR congres* OR abstract* OR book* OR chapter* OR dissertation abstract*).pt. NOT (case reports/ OR (case report*).ab,ti.)

Cochrane CENTRAL

((asthma*):ab,ti) AND ((child* OR kid OR kids OR schoolchild*):ab,ti) AND ((questionnaire* OR Control NEXT Test* OR gina OR global NEXT initiative* OR checklist* OR check NEXT list* OR screening NEXT tool* OR act OR acq OR instrument* OR scoring OR scale OR ((measure* OR assess* OR determine* OR tool*) NEAR/3 control*)):ab,ti) AND (((((compar*) NEAR/10 (questionnaire* OR Test* OR gina OR checklist* OR check NEXT list* OR tool* OR method* OR intermethod* OR stud* OR assess* OR act OR acq OR instrument* OR scoring OR scale OR global NEXT initiative* OR effect*)) OR (clinical* NEAR/3 value*) OR (measurement* NEAR/3 (propert* OR characterist*))):ab,ti) OR ((((primary OR family OR general) NEAR/3 (care OR pract* OR medicine* OR physician* OR doctor*)) OR gp OR gps):ab,ti))

Web of science

TS=(((asthma*)) AND ((child* OR kid OR kids OR schoolchild*)) AND ((questionnaire* OR Control-Test* OR gina OR global-initiative* OR checklist* OR check-list* OR screening-tool* OR act OR acq OR instrument* OR scoring OR scale OR ((measure* OR assess* OR determine* OR tool*) NEAR/2 control*))) AND (((((compar*) NEAR/10 (questionnaire* OR Test* OR gina OR checklist* OR check-list* OR tool* OR method* OR intermethod* OR stud* OR assess* OR act OR acq OR instrument* OR scoring OR scale OR global-initiative* OR effect*)) OR (clinical* NEAR/2 value*) OR (measurement* NEAR/2 (propert* OR characterist*)))) OR ((((primary OR family OR general) NEAR/2 (care OR pract* OR medicine* OR physician* OR doctor*)) OR gp OR gps)))) AND DT=(article)

Google scholar

asthma adolescents|infants|newborns questionnaire|questionnaires compare|compares|comparison|"Primary Health|care|Healthcare"|"general|family practice|practitioners|physicians"

Table 6. Quality criteria for measurement properties of the included questionnaires.

Questionnaire	Content validity	Internal consistency	Criterion validity	Construct validity	Agreement	Reliability	Responsiveness	Floor or ceiling effect	Interpretability
**RCP3Q[I]**	?	?	+*	0	0	0	0	-	?
**APGAR[II]**	+	0	+	0	0	0	0	0	?
**ACQ[III]**	+	0	0	?	?	?	?	0	?
**RCP3Q[IV]**	?	0	0	?	0	-	0	-	?
**VAS[V]**	0	0	-	0	0	0	0	-	?

Rating: + = positive; ?=intermediate; -=poor; 0=no information available.

*RCP3Q≥1 results in – and RCP3Q≥2 results in +

RCP3Q Royal College of Physicians three questions
C-ACT Childhood Asthma Control Test
ACT Asthma Control Test
APGAR Activities Persistent triGGers Asthma medications Response to therapy
ACQ Asthma Control Questionnaire
NAEPP National Asthma Education and Prevention Program
VAS Visual Analog Scale
I= Adrews et al. 2018; II=Rank et al. 2014; III=Juniper et al. 2010; IV=Thomas et al. 2009; V=Halterman et al. 2006
